# Review and consensus on pharmacogenomic testing in psychiatry

**DOI:** 10.1017/S0033291724002095

**Published:** 2024-11

**Authors:** Mark Faiz, Sanjida Ahmed

**Affiliations:** Personalized Prescribing Inc., Toronto, ON, Canada

Dear Editors,

We have carefully reviewed the article titled ‘The emergence, implementation, and future growth of pharmacogenomics in psychiatry: a narrative review’ by Chad Bousman et al. published in *Psychological Medicine*, on 29 September 2023 (Bousman et al., 2023a, 2023b). We commend the authors for their comprehensive review and wish to contribute to the discourse surrounding pharmacogenomics.

The introductory narrative of the article aptly highlights that PGx involves the analysis of variations in the genes responsible for pharmacokinetics (i.e. absorption, distribution, metabolism, and elimination) as well as pharmacodynamics (i.e. target receptors and signaling pathways); these genetic variants largely indicate whether a medication will work effectively or be well-tolerated. In addition, they emphasized that the CPIC (Clinical Pharmacogenetics Implementation Consortium) and FDA (Food and Drug Administration) advocate for the use of PGx testing, specifically testing *CYP2D6* and *CYP2C19* genes to guide dosing of antidepressants, antipsychotics, and atomoxetine (Bousman et al., 2023a, 2023b).

While we agree with Bousman et al.'s conclusions that testing for *CYP2D6* and *CYP2C19* is important, testing for these genes alone is not sufficient to determine overall drug efficacy, nor is it cost-effective. Data suggest that almost 90% of all ethnicities have either extensive or intermediate function of the two liver enzyme genes predominantly associated with normal/moderate metabolism and clearance of antidepressants, while poor metabolizers and rapid metabolizers are a small minority of the population, with the exception of Oceanians (CPIC, 2023). Furthermore, guidelines for prescribing antidepressants recommend initiating treatment at low doses and slowly titrating to a therapeutic level (CPIC, 2023); treating practitioners intuitively recognize the poor and rapid metabolizers within a few days to weeks of initiation, diminishing the value of pharmacokinetic testing.

Unlike pharmacokinetics, pharmacodynamics involves a polygenic analysis rather than a one-gene drug analysis (Fabri, 2017). PGx tests are not complete without a robust analysis of pharmacodynamics. Unfortunately, apart from briefly mentioning the incorporation of PGx markers in the pharmacodynamics process, Bousman et al. do not discuss the specifics of implementation in their review.

Although limited research exists on how polygenic pharmacodynamics influences antidepressants, there is extensive knowledge available on drug mechanism of action and genetic activity of the SNPs variants that are involved in the drug pathway.

Given the current knowledge, we suggest that pharmacodynamic algorithms can be developed using a polygenetic analytical approach. Such algorithms can be of additive value to CPIC guided pharmacokinetics, in order to provide a comprehensive pharmacogenomic test that assesses likelihood of drug efficacy.

While the science behind this proposed polygenic analysis may not be precise, its purpose is to provide healthcare providers with insights into their patients' complete pharmacogenomic profiles, which can ultimately assist with making prescribing decisions. Undoubtedly, this approach offers a more informed alternative to prescribing in the absence of genetic information.
